# Flavonoids from *Clerodendrum* genus and their biological activities

**DOI:** 10.5599/admet.2442

**Published:** 2024-12-10

**Authors:** Meiske Naomi Mamuaja, Tati Herlina, Rymond Jusuf Rumampuk, Iman Permana Maksum, Yaya Rukayadi

**Affiliations:** 1 Department of Chemistry, Faculty of Mathematics and Natural Science, Universitas Padjadjaran, Sumedang 45363, Indonesia; 2 Department of Chemistry Universitas Negeri Manado, Tondano 95618, Indonesia; 3 Department of Food Science Universiti Putra Malaysia, Malaysia

**Keywords:** Traditional medicine, secondary metabolites, isolation, bioactivities

## Abstract

**Background and purpose:**

Many studies have been performed to identify new sources, their optimal isolation, and the biological activities of flavonoids due to nutraceutical, pharmaceutical, and cosmeceutical properties.

**Experimental approach:**

This review describes the method for flavonoid isolation and characteristic from the *Clerodendrum* genus and their biological activities with the indication of the most active ones. To perform a comprehensive review, a thorough literature review using Google Scholar, Scopus, and Science Direct was performed with keyword alone or in combination with other words.

**Key results:**

The isolation and identification of flavonoids from the Clerodendrum genus have revealed a variety of compounds using various methods. Various studies conducted *in vivo*, *in vitro* and *in silico* also reported bioactivities of these flavonoids.

**Conclusion:**

Several factors determine the flavonoid content in the *Clerodendrum* genus, among others, the different parts of the plant, extraction techniques, and solvent combination used. Isolated flavonoids also show significant biological activities, such as antioxidant, anti-inflammatory, antimicrobials, antidiabetic, anticancer, anti-tyrosinase, and neuroprotective agents.

## Introduction

*Clerodendrum* genus has been used as ornaments in celebration and traditional medicine in India, China, Korea, Japan, Thailand, Indonesia, and Africa [1,2]. Linnaeus was the first to identify *Clerodendrum* genus and later named it *Clerodendrum informatum* in 1753 in India. At first, the genus was put into Family *Verbenaceae* and later after a phylogenetic analysis through molecular data, it was included in Family *Lamiaceae* in 1990 [3,4]. The word *Clerodendrum* itself comes from Greek ‘kleros’ (destiny) and ‘dendron’ (tree), which might relate to two definitions of the use of species of the genus at that time; some of the species are believed to have curing properties, and others have toxic properties [5].

*Clerodendrum* genus has at least 500 species and grows in tropical and warm regions of Africa, eastern and southern Asia, and also in America and the northern part of Australia [6]. They are evergreen shrubs, lianas, small and woody vines [7]. The members of the *Clerodendrum* genus have been reported for traditional medicines, for example, *Clerodendrum trichotomum* for rheumatic and headaches [8], *Clerodendrum serratum* for asthma and malaria [9] and inflammation [10], *Clerodendrum inerme* for hepatitis and antidote [11], *Clerodendrum colebrookianum* for hypertension [12] and *Clerodendrum philippinum* for anti-diabetic [13]. Based on these therapeutic properties, *Clerodendrum* members have been studied for more than decades, resulting in extraction, isolation, purification, and identification of some compounds, including flavonoids and their glycosides, terpenoids, phenylethanoid glycosides, steroids and their derivates, cyclo-hexyl ethanoic and cyanogenic glycosides [14]. There are more than 300 compounds have been isolated and identification from the *Clerodendrum* genus, where pharmacological studies indicate that the crude extract and several monomers have various biological activities such as antioxidants, antidiabetic, anti-inflammatory, anticancer, antimicrobe, antihypertension, anti-obesity, antidiarrhea, liver protection, improving memories and neuroprotective [15].

**Figure 1.**
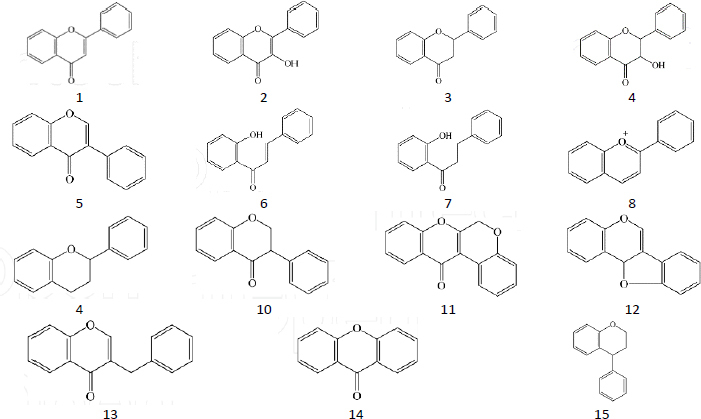
Main structure of flavonoid: flavone (1), flavonol (2), flavonone (3), flavanonol (4), isoflavone (5), chalcone (6), dihydrochalcone (7), anthocyanidin (8), aurone (9), isoflavanone (10), rotenoid (11), pterocarpan (12), homoisoflavanone (13), xanthone (14) and neoflavonoid (15).

Flavonoids are a group of secondary metabolites abundant in plants and represent the third largest group of natural products after alkaloids and terpenoids [16]. Flavonoids have a polyphenolic structure and are produced for plant growth and defines mechanisms [17] by acting as attractants for pollinators, sunscreen to protect against solar radiation, antimicrobial and antiherbivore [18]. The basic structure of flavonoids is C_6_-C_3_-C_6_, where two aromatic rings, A and B, are connected by a unit of three carbon atoms [19]. Flavonoids are classified based on the connection model of ring A and ring B, the position of ring B connection, the oxidation level of ring C substructure and the degree of polymerization, where the main structure is as in Figure 1 [20]. Variations in the structure of these flavonoids produce several biological activities that are important for medicine [21].

Research shows that flavonoids have nutraceutical, pharmaceutical, medicinal and cosmetic functions due to their ability to act as antioxidants, anti-inflammatory, antimutant and anticarcinogenic [22]. At the cellular level, they combine to modulate key cellular enzyme functions [23]. The flavonoid content in *Clerodendrum* genus has been reported in several studies with varying bioactivities [24,25]. Therefore, this review examines the members of the *Clerodendrum* genus and their flavonoid presence, highlighting their isolated methods and their biological potential in the area of the most often studied activities, such as antioxidant, antidiabetic, anti-inflammatory, antitumor, and others.

## Isolation and characterization of flavonoids from *Clerodendrum* genus

Based on flavonoids potential for human health, several techniques and conditions for the isolation of this compounds have been implemented [26]. The common procedure for the isolation process involves plant preparation, extraction and fractionation, and purification [27]. The type of solvent and its polarity, solvent combination, and the ratio of liquid-solid have a significant impact on the process [28]. Various solvents, methods and parts of the plants used for isolation from the *Clerodendrum* genus are given in Tables 1-6, based on the *Clerodendrum* species.

**Table 1.** Flavonoid Isolated from *Clerodendrum paniculatum* Linn

Material	Solvent and Extraction Method	Isolated flavonoid	Activity	Ref.
Roots	Maceration with ethanol; fractination, and ethyl acetate fraction was subject to column chromatography with DCM:MeOH = 7.5:2.5	Quercetin	-	[30]
Flower	Maceration with alcohol, no available data for CC solvent	Quercetin	Antioxidant Hepatoprotective	[31]
Leaves	Maceration with n-hexane, fractionation with ethyl acetate and ethanol	Apigenin scutellarin	Antibacterial	[32]

**Table 2.** Flavonoid Isolated from *Clerodendrum inerme*

Material	Solvent and extraction method	Isolated flavonoid	Activity	Ref.
Leaves	Extraction with 95 % ethanol, partition with hexane, chloroform and butanol. CC with silica gel for chloroform using MeOH/CH_2_Cl from 0 %, 1 %, 2 %, 10 % to get 9 fractions; a crystal of fraction 7 is the subject for NMR compound identification	Hispidulin	Preventing hyperdopaminergic activity	[40]
Leaves and stems	Maceration with ethanol, fractionation, CC for ethyl-acetate with chloroform = 25:75 weight ratio	Apigenin, Salvigenin, acacetin	-	[41]
Leaves and stems	VLC n-hexane: ethyl-acetate, CC with n-hexane: ethyl-acetate	5-hydroxy-6,7, 40-trimethoxyflavone	Antioxidant, anti-inflammatory	[42]

**Table 3.** Flavonoid isolated from *Clerodendrum phlomidis* leaves

Material	Solvent and Extraction Method	Isolated flavonoid	Activity	Ref.
Leaves	Fractionation with chloroform, hexane and ethyl acetate; chloroform extract was subject to column chromatography with hexane-ethyl acetate as eluent; found 12 fraction, fraction 5 was subject to re-column with combination of chloroform: ethyl acetate = 9:1. The final fraction was subject to HPLC and NMR checked.	Pectolinaringenin	Natural mosquito larvicidal agent	[52]
Leaves	Defatted by PE; extracted with methanol; partition in butanol. The butanol extract was subject for CC with chloroform: methanol; the flavonoid fraction was re-column using the same solvent; and found the compound in combination of 85:15 % and 80:20 %, chloroform: methanol	Pectolinaringenin unnamed flavonoid 1 and 2	-	[53]

**Table 4.** Flavonoid Isolated from *Clerodendrum petasites*

Material	Solvent and extraction method	Isolated flavonoid	Activity	Ref.
Aerial	Maceration with 96 % ethanol, separated in ethyl-acetate : methanol : water (43:22:35), subsequent fraction by centrifugal partition chromatography (CPC) using chloroform : methanol : n-propanol : water (45:60:10:40). Fraction than subject to TLC using ethyl-acetate : formic acid : acetic acid : water (100:11:11:27) Active fraction was then subject to NMR	Hispidulin	Treatment for asthma	[65]
Root	Maceration with 80 % ethanol, partitioned with water, ethyl acetate and dichloromethane then determined by HPLC	Hispidulin Hesperetin Hesperidin	Inhibits SARS-CoV-2 spike protein	[67]
Leaves stem, root	Extracted with methanol and subjected to HPLC with the combination of acetonitrile and acetic acid for the mobile phase	Quercetin Hispidulin	Anti- inflammatory	[68]
Aerial parts	Maceration with 80 % ethanol, partitioned with water, butanol, ethyl acetate and petroleum ether; the ethyl acetate and butanol fractions are subject to column chromatography and examined by NMR	Hispidulin Apigenin Luteolin Nepetin	Skin treatment	[69]

**Table 5.** Flavonoid Isolated from *Clerodendrum volubile*

Material	Solvent and extraction method	Isolated flavonoid	Activity	Ref.
Leaves	Methanol extract was partition with n-hexane, DCM, ethyl-acetate and butanol. DCM fraction was subject to CC with n-hexane : DCM (92.5:7.5)	Pectolinaringenin	Antioxidant, anti-inflammatory	[74]

**Table 6.** Flavonoid isolated from *Clerodendrum glandulosum* leaves

Material	Solvent and Extraction Method	Isolated flavonoid	Activity	Ref.
Leaves	Maceration with methanol 95 %; the extract was then subject for HPLC analysis	Scutellarin, luteolin, apigenin	Antioxidant Antidiabetic	[83]
Leaves	Maceration with methanol 80 fractionation to hexane, followed by chloroform, the residual brown aqueous phase was considered as the polyphenol-rich fraction that subject for HPLC analysis	Apigenin	Antioxidant Antidiabetic	[84]

### *Clerodendrum paniculatum* Linn

Traditionally, *Clerodendrum paniculatum* Linn (pagoda flower) has been used for medicine in many Asian and African countries, especially for the treatment of fever, asthma, hypertension, rheumatic, microorganism infection, leprosy and tumours [29]. Leena and Aleykutty [30] isolated quercetin (3,3’,4’,5,7- pentahydroxy flavone) from the roots of this plant, while Koppilakal *et al.* [31] isolated it from the flower (Table 1) and reported antioxidant activity and hepatoprotective effects of the flower in white male rats induced with CCl_4_. Using LC-MS/MS, Pertiwi *et al.* [32]reported the presence of several flavonoids in the ethyl acetate extract. These flavonoids include apigenin, apigenin 7-O-glucuronide, 6-O-methylscutellarin, apigetrin (7-(β-D-glucopyranosyloxy)-4′,5-dihydroxyflavone), 4-Coumaric acid, 7-Hydroxycoumarine and scutellarin. They also reported the strong antibacterial activity of the plant leaf extract.

Research on *Clerodendrum paniculatum* Linn has elicited a wide range of biological activities. Hafiz *et al.* [33] reported the antioxidant (*IC*_50_ value of 27.73376 μg/ml) and anti-inflammation (50 mg/kg BW) activities of leaves ethanol extract on white male rats, while Hedge *et al.* reported the antioxidant activity from methanol extract and the antidiabetic activity through inhibition of α-amylase enzyme [34]. The antidiabetic activity was also reported of the chloroform extract, with an *IC*_50_ value of 158.396 μg/ml for α-amylase inhibition test and an *IC*_50_ value of 113.122 μg/ml for the α-glucosidase inhibition test [35]. The *in vivo* test on diabetic-induced rats in this research found a significant decrease in blood glucose, while the *ex vivo* tests using the rat hemidiaphragm isolation method showed a significant glucose uptake value. The flower ethanol extract was also reported for the capability to reduce blood glucose, improve lipid metabolism and body weight in diabetic-induced rats [36].

The anti-anxiety activity in female albino mice (*Wistar* strain) was reported by Priyanka [37] from the ethyl acetate extract of the stems. This activity was associated with the flavonoid content, which modulates or inhibits gamma-aminobutyric acid (GABA) in the nervous system. Furthermore, a study by Sundaraganapathy *et al*. [38] on male Swiss white mice injected with Dalton's Lymphoma ascites (DAL) cancer cells showed anticancer activity from the root extract.

### Clerodendrum inerme

*C*lerodendrum *inerme* is known by the local names *Melati laut* (sea jasmine), *lamburung meit*, *gambir laut* (sea gambir) and *genje* in Indonesia, and is widespread in South China, India, Southeast Asia and North Asia. Traditionally, this plant is widely used to treat rheumatic pain, skin diseases, venereal diseases, wounds, fever, cough, dysentery and more [39]. Isolation carried out on this plant found a number of flavonoids such as hispidulin [40], apigenin, salvigenin and acacetin [41] and 5-hydroxy-6,7,40-trimethoxyflavone [42] with anti-depression, antioxidant and anti-inflammatory activity (Table 2). Huang *et al.* [40] reported that the isolated compound can alleviate methamphetamine-induced hyperlocomotion, thus preventing hyperdopaminergic disorders, while Ibrahim *et al.*[41], using formalin-induced rats, reported the antioxidant and anti-inflammatory activities due to the possibility of the isolated flavonoid modifying free radicals and reactive nitrogen species, and inhibiting prostaglandin synthase enzyme.

Yankanchi and Koli [43] reported the anti-inflammatory activity of methanol extract of *C. inerme* leaves against male white rats induced with acetic acid. Research by Nindatu *et al.* [44] showed a decrease in malaria parasite density in patients but did not cause toxic effects on the liver and kidneys as indicated by unchanged levels of SGOT, SGPT, urea and creatinine. Toxicity tests by Khan *et al.* [45] also showed non-toxic properties with high antioxidant activity. Fan *et al.* [46] reported that the use of juice from the leaves of the plant was able to relieve intractable motor tic disorder in a 13-year-old Tourette syndrome (TS) patient without causing side effects. *In vivo* tests on white male mice that were injected with methamphetamine and NMDA channel blockers to mimic TS showed that *Clerodendrum inerme* leaf extract was able to suppress hyperlocomotion and inhibit pre-pulse inhibition (PPI), a condition also experienced by people with psychiatric disorders such as schizophrenia, attention deficit hyperactivity disorder (ADHD) and obsessive-compulsive disorder - OCD [47].

### Clerodendrum phlomidis

*Clerodendrum phlomidis* is included in the traditional Ayurvedic, Unani and Siddha medicinal systems in India both as a single drug and in combination with other plants [48]. Ethnomedicinally, this plant is used to treat syphilis and gonorrhea, as well as children affected by measles [49], rheumatism [50], digestive disorders, acidity, gas diarrhea, liver tonic and ingredients for many pain relief massage oils [51]. Isolation carried out on the leaves of this plant showed the presence of pectolinaringenin and proposed the compound as a natural mosquito larvicidal agent due to its effectiveness in reducing two mosquito larvae, *Culex quinquefasciatus* Say and *Aedes aegypti* L [52]. Pectolinaringenin was also isolated by Bharitkar *et al.*[53], along with two new flavonoid glycosides (Table 3). The antibacterial activities of *Clerodendrum phlomidis* were reported by Yadav *et al.*[54], using the BACTEC radiometric susceptibility assay against *Mycobacterium tuberculosis* H37Rv (ATCC 27294), and Vaghasiya and Chanda [55] using the agar disc diffusion method for *Staphylococcus epidermidis* inhibition. Research by Dhanabal *et al.* [56] using alloxan-induced diabetic rats shows the hypoglycemic and hypolipidemic activities of leaf ethanol extracts, while Chidrawar *et al.* [57]reported the anti-obesity activity of root methanol extract by reducing the level of circulating lipid and adipocyte diameter, resulting in the decrease of body weight in C57BL/6J rats. The antiarthritic activity of the plant leaves was reported by Patel *et al.* [58]using Freund’s complete adjuvant (FCA)-induced rats. Research on this plant also reports the immunemodulatory activity [59], neuroprotective agent [60], and antidiarrheal activity [61].

### Clerodendrum petasites

*Clerodendrum petasites* (English name: one root plant) is widespread in Thailand, Vietnam, China, India, Malaysia and Sri Lanka [62]. In Thailand, this plant is included in the traditional medicine mixture "Ben-Cha-Lo-Ka-Wi-Chian Remedy" as an antipyretic [63], antiasthma and anti-inflammatory [64]. The isolation of *Clerodendrum petasites* identifies a number of flavonoids and reports various bioactivities (Table 4). Hazekamp *et al.* [65] isolated hispidulin from the aerial part and reported the relaxing effect on the tracheal smooth muscle of guinea pigs from the plant ethanol extract. This relaxing effect is also reported by Hasriadi *et al.* [66] through the nociceptive pain test, where the administration of the extract to experimental mice was able to alleviate pain-like behaviours, such as thermal nociceptive pain, abdominal constriction, neurogenic, and inflammatory pain.

Using the root ethyl acetate extract, Asjri *et al.* [67] isolated three flavonoids: hispidulin, hesperetin, and hesperidin. The research also tested for chronic inflammation using A549 lung cells and found that the ethyl acetate extract and hesperetin can significantly inhibit the Spike S1-induced inflammatory gene expressions (NLRP3, IL-1*b*, and IL-1). They proposed the use of *Clerodendrum petasites* extract and hesperetin in the development of supportive therapies for the prevention of COVID-19-related chronic inflammation. Kwuansawat *et al.* [68] isolated quercetin and hispidulin from leaves, stems and roots and reported the anti-inflammatory activities of the methanol extracts by *in vitro* testing using RAW 264.7 cells. The anti-inflammatory activities were also reported by Panthong *et al.* [70] using ear edema rats induced by ethyl phenylpropiolate.

Isolation conducted by Thitilertdecha *et al.* [69] on aerial parts identified a number of flavonoid aglycones, including nepetin, luteolin, apigenin, naringenin, hispidulin, hesperetin, and chrysin. Using a pig skin model, the research shows that hispidulin and nepetin were able to penetrate the skin, making skin treatments possible. An *in vivo* test of lotions and creams with a mixture of *C. petasites* extract showed that hispidulin and nepetin were mostly absorbed by the stratum corneum 6 hours after being applied to human skin [71].

### Clerodendrum volubile

*Clerodendrum volubile*, known as a white butterfly, is a shrub-like climber native to Africa, widely distributed in warm temperate and tropical regions of the world, and is used for ornament, as a food ingredient, and in traditional medicine [72]. The plant has long been used traditionally to treat arthritis, diabetes, dropsy, gout rheumatism, swellings, oedema, as an analgesic, pregnancy tonic, anti-abortifacients, and sedatives [73]. Table 5 summarizes the plant parts, extraction methods, flavonoids, and their bioactivities isolated from *C. volubile.*

Erukainure *et al.* [74] isolated pectolinarigenin for the first time from the leaf extract. The research also shows that the DCM fraction of *Clerodendrum volubil*e shows potent immunomodulatory activity by inhibiting T-cell proliferation and modulating respiratory oxidative burst in phagocytes. A study by Ugbaja *et al.* [75]on rats induced with arsenic shows the hepato-reno protective effects of leaf extract through reducing oxidative stress and increasing antioxidant molecules/enzymes, singly or combined.

*Clerodendrum volubile* has been reported for its antiproliferative activity. Erukainure *et al*. [76] reported the antiproliferative activity of fatty acids from leaves against MCF-7 human breast cancer cell lines. The fatty acids considerably inhibited cell growth, arrested G0/G1 phase by down-regulating the gene expression (MMP-9) and mitigated oxidative stress in MCF-7 cell lines. Another study by Erukainure *et al.* [77] on the antiproliferative effect of the dichloromethane leaf extract revealed that the extract exhibited cytotoxic effects against human embryonic kidney (HEK293) cells. A concurrent increase in proinflammatory biomarkers, reduction of antioxidative biomarkers, and ATP depletion led to cell apoptosis. Saheed *et al.* [78] also reported an antiproliferative effect from the leaf methanol extract against prostate cancer (PCa) cells. The extract was able to suppress the clonogenic potential of PCa cells in a colony and 3-(4,5-dimethylthiazol-2-yl)-2,5-diphenyl-2H-tetrazolium bromide (MTT) assays. An increase in the levels of cyclin-dependent kinase inhibitor p21 signified the modulation of the cell cycle machinery, while a concentration-dependent cleavage of Poly (ADP-ribose) polymerase (PARP) and Caspase 3 was observed through the western blot analysis of the extract-treated cells.

The antioxidant potential of *Clerodendrum volubile* has been reported by many studies. Ogunwa *et al.* [79] reported the aqueous extract using metal chelating, reducing power, 2, 2′-azino-bis (3-ethylbenthiazoline-6-sulphonic acid (ABTS), DPPH, superoxide anion and hydrogen peroxide scavenging assays and showed very good antioxidant activity in all the tested assays. Adefegha and Oboh [80] reported a similar effect of the aqueous extract with standard antioxidants such as ascorbic acid, trolox and EDTA, by using iron chelating, DPPH radical, superoxide ion, hydrogen peroxide, ABTS radical, hydroxyl radical scavenging activities and ferric ion reducing properties assays.

### Clerodendrum glandulosum

Clerodendrum glandulosum (India's traditional name: glory bower) has been known for its use in “Ayurvedic Medicine”, one of the oldest medical systems from India [81]. People from the northeast region of India use the dried leaves of this species as a traditional remedy for obesity, hypertension and diabetes [82]. Deb et al. [83] identified scuttelarin, luteolin and apigenin from the leaf of this plant (Table 6). The research also reported the antioxidant activity using DPPH, ABTS, FRAP, phospho-molybdenum reduction, and SOD assay, and the inhibition activity of metabolic enzymes, such as α-glucosidase, α-amylase, pancreatic lipase, xanthine oxidase, and angiotensin-converting enzyme. Using a polyphenol-rich fraction for extraction, Kound *et al.* [84] isolated apigenin and reported the potency of antioxidant (*IC*_50_ of DPPH = 32.45 μg/mL; ABTS = 39.08 μg/mL) and antidiabetic (*IC*_50_ = 2.18 μg/mL for aldose reductase inhibition) properties from the fraction. The *in vivo* test shows the reduction in blood glucose levels and increase in plasma insulin in a diabetic rat model, and the *in silico* test shows the interaction of hydrogen bond between apigenin and amino acid residues of α-amylase, α-glucosidase, and aldose reductase enzymes.

Extracts obtained from the leaves of *Clerodendrum glandulosum* have been reported to have antioxidant, hepatoprotective, anti-inflammatory, cardioprotective, hypolipidemic, anti-obesity, anti-hyperglycemic and antidiabetic properties [85]. It also prevents adipocyte differentiation and visceral adiposity by downregulating peroxisome proliferator-activated receptor γ (PPAR-γ) related genes and leptin expression, thus validating its traditional therapeutic use in controlling obesity [86]. The methanolic extract of this plant was assayed for its free radical scavenging potential using different in vitro assays and showed strong antioxidant activity [87]. Regarding metabolic disorder, the freeze-dried extract was reported to be able to regulate plasma lipids in hyperlipidemia rats [88]. The hypolipidemic effects on a rat model of hyperlipidemia were studied by Jadeja *et al.* [89], which showed a decrease in body weight (9.6 %), plasma total cholesterol (15.63 %), triglyceride (42.99 %), phospholipids (13.91 %), LDL-C (81.36 %), and VLDL-C (43 %) along with an increase in HDL-C (52.84 %).

## Biological activities of isolated flavonoids

### Quercetin

**Figure 2.**
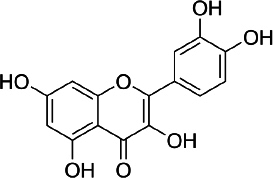
Quercetin

Quercetin (C_15_H_10_O_7_, Figure 2) is a flavonoid in fruits and vegetables. It has unique biological properties that can improve physical and mental health and reduce the risk of infection [90]. Quercetin has diverse biological activities, such as antioxidant [91,92], antidiabetic [93], cardioprotective [94], anti-obesity [95] antihypertension [96], anti-Alzheimer [97], antitumor [98] and anti-hyperpigmentation [99].

The antioxidant properties of quercetin occur due to its ability to capture free radicals and bind transition metal ions [100]. The presence of the 3',4'-diphenolic group in quercetin allows this flavonoid to be effective in superoxide anion scavenging activity [101].

Research shows that quercetin will be produced in white blood cells and the liver shortly after consuming vegetables rich in quercetin [102], where quercetin is able to prevent damage to red blood cell membranes due to smoking [103]. Several studies have shown that quercetin has hepatoprotective activity in mice induced with a high-fat diet [104], reducing oxidative stress caused by hyperglycemia and diabetes by modulating carcinogenic signalling pathways [105].

Several studies have shown the antidiabetic mechanism of quercetin *in vitro* and *in vivo* through increasing pancreatic islet regeneration and possibly increasing insulin release in STZ diabetic rats [106], reducing oxidative stress and protecting pancreatic β-cells [107], improving liver and kidney function by restoring cell proliferation through inhibiting CDKN1A gene expression [108], increasing adiponectin secretion by PPAR-γ independent mechanism, and reducing fasting glucose and HbA1c through decreasing intestinal maltose activity [109]. In their research, Ahmet *et al.* showed that quercetin accelerated wound healing in diabetic and non-diabetic mice through the mechanism of reducing proinflammatory cytokines [110], inhibited the secretion of cytochrome P450 2E1 (CYP2E1) during the development of diabetes, thereby preventing oxidative damage in the liver [111] and also provided neuroprotective effects through the mechanism of preventing acetylcholinesterase (AChE) activity in the brains of diabetic mice [112]. In the case of anti-obesity, quercetin is reported to have the ability to stimulate hepatic mitochondrial oxidative metabolism through the induction of *heme oxygenase*-1 (HO-1) in the nuclear factor-related erythroid factor 2, Nrf-2 pathway [113]. In studies of cardioprotective activity, quercetin was reported to work in post-traumatic reversal reactions of cardiac dysfunction by reducing cardiomyocyte apoptosis, thereby suppressing the increase in tumour necrosis factor (TNF) alpha, reactive oxygen species (ROS) and Ca^2+^ production [114], as well as an alternative treatment for ischemia-reperfusion injury (IRI) by inducing blood vessel dilation through inhibition of endothelin-1 receptors, increased stimulation of nitric oxide (NO) and activation of calcium channels [115]. For antihypertensive activity, quercetin works to inhibit the activity of the cytochrome P450 (CYP) 4A enzyme and the soluble epoxide hydrolase (sHE) enzyme, the two main enzymes of arachidonic acid metabolism in the kidneys which regulate blood pressure [116]. In *in silico* research using molecular docking, quercetin was able to bind angiotensin-converting-enzyme (ACE), an enzyme responsible for regulating blood pressure with an optimal binding energy of −35.564 kJ/mol [117].

Having a neuroprotective activity, quercetin works to inhibit cholinesterase (ChE) activity, thereby restoring the balance of acetylcholinesterase (AChE) and butyrylcholinesterase (BChE) in brain tissue [118]. Studies in mouse models of Alzheimer's showed that quercetin reduced extracellular β-amyloidosis plaques, tauopathy, astrogliosis and microgliosis in the hippocampus and amygdala of mice, which are characteristic of Alzheimer's [119]. Quercetin induces apoptosis through activation of the mitochondrial pathway (caspase cascade) and by inhibiting signals in the human hepatoma cell line - HepG2 [120], disrupts the Akt/PKB pathway by inhibiting the proliferation process and induces apoptosis [121], increases TNF-related cytotoxicity apoptosis-inducing ligand (TRAIL-anticancer drug) by activating caspases and inhibiting Akt phosphorylation [122] and induces the apoptotic pathway in MCF-7 cells [123]. Structurally, the mechanism of action of quercetin is the inhibition of the DNA topoisomerase I and II enzymes, which play a role in cleavage in the proliferate phase, through substitution of the keto group C-4 and substitution of the hydroxyl groups of ring A and ring B at positions C-3, C-5, C-7, C3' and C4' [124].

Studies also reported the ability of quercetin to inhibit tyrosinase, an enzyme that plays a role in the synthesis of melanin [125]. Controlling tyrosine activity will lead to the treatment of hyperpigmentation disorders in mammals and enzymatic browning of fruits and fungi [126]. An *in vitro* study showed that quercetin could significantly inhibit both the monophenolase and diphenolase activity of tyrosinase and inhibited the formation of dopaquinone in a reversible competitive manner with an *IC*_50_ value of (30.8±7.4) 4mol/L [125] and (44.5±1.3) 3mol/L [127]. *In silico studies* through molecular docking suggested the inhibition activity of quercetin due to the catechol structure (3’,4’ dihydroxy groups in B ring) of quercetin that chelated copper in the active site of tyrosinase resulting in the blocking access of the substrate L-DOPA. The report showed the non-hydrogen bonding interaction with various amino acid residues, including Gly281, Ser282, Met280, His263, Phe292, Val283, His61, Ala286, His85, His259, Phe264, Asn260, Met257 and Val248 [127], while Fan *et al.* [128] reported this bonding occurs on Cys83 and His85. Another study by Park *et al.* [129] reported that quercetin-7--O-α-L-rhamnoside, a quercetin glycoside, inhibits tyrosinase activity and melanogenesis in α-MSH plus IBMX-stimulated B16F10 melanoma cells. Docking simulation revealed hydrogen bonding of this flavonoid with amino acid residues His85, His244, Thr261, and Gly281 of tyrosinase.

Despite its diverse biological activities, the use of quercetin is limited by its low level of solubility, which affects its bioavailability [130]. Several studies have been carried out to increase the bioavailability of this compound, including the combination of quercetin with insulin, which can increase bioavailability by 20 % [131], encapsulating quercetin with lecithin-chitosan nanoparticles [132], encapsulating with lipid nanoparticles [133], using rice bran protein as an emulsifier [134], co-crystallization with nicotinamide [135], and by attaching a sugar group to the 3-OH position of quercetin in order to increase the whitening effect [136].

### Apigenin

**Figure 3.**
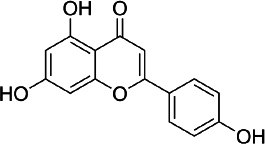
Apigenin

Apigenin (APG, 4′,5,7-trihydroxyflavone - C_15_H_10_O_5_, Figure 3) attracted attention for the first time in the 1960s when this compound was found to suppress the release of histamine from cells of white basophils and exhibited bronchial dilating effects on the lungs [137]. A number of studies show the ability of these flavones to inhibit and stop cell proliferation in several types of cancer, such as pancreatic, colon, liver, blood, lung, prostate, breast, thyroid, skin and neck [138-140]. In addition, apigenin is also reported to have antioxidant [141], and anti-inflammation [142].

Currently, the development of research on apigenin therapy is being focused on its use to reduce chemotherapy resistance of various anticancer drugs by targeting several signalling pathways at the cell/molecular level [143]. Research on the effect of apigenin at the cellular level on prostate cancer using transformed human prostate epithelial cells and various prostate cancer cells (RWPE-1 cells and prostate cancer LNCaP, PC-3 and DU145 cells) *in vivo* and *in vitro* found that apigenin accumulates in the nuclear matrix and binds to DNA, thereby reducing oxidative DNA damage and apoptosis [144].

Another mechanism was also reported by Sukhla and Gupta [145], where apigenin accumulated in cells has the potential to interfere with androgen receptor signalling and inhibit androgen-responsive genes. In breast cancer research, apigenin was reported to be able to block the development of progestin-dependent BT-474 breast cancer cell (BCC) xenograft tumours in female mice [146]. In addition, apigenin is able to reduce cell proliferation through modulation of mitogen-activated protein kinase (MAPK), phosphoinositide 3-kinase (PI3K-Akt) [147,148]. This MAPK modulation is also key to the anti-inflammatory activity of apigenin [149], while Kang *et al.* [150] demonstrated apigenin's regulation of the production of TNF-α, IL-6, IL-8, and GM-CSF in HMC-1 cells. A molecular docking study shows the interaction between apigenin and some genes related to papillary thyroid carcinoma with the binding energy range from −31.4636 to −18.7025 kJ/mol [151].

Zhang *et al.* [152] reported the whitening effect of apigenin through *in vitro* testing using melanin production and tyrosinase activity assays. They propose that the higher rate of inhibitory activity is due to the presence of 7 and 4’ hydroxyl groups in this flavonoid. An *in vitro* test was also reported by Karaoglan *et al.*[153], where apigenin showed a tendency to inhibit tyrosinase activity by 49.36±0.24 %. Molecular docking simulations showed a hydrogen bond between the hydroxyl group of the benzopyran ring and the carbonyl group of Met280, as well as a hydrophobic interaction with residues of Val248, Phe264, Met280, Val283, Ala286, and Phe292. Additionally, polar interactions were observed with His61, Hid85, Ser282, His263, Asn260, and His259. However, an *in vivo* study report by Chauhan *et al.* [154] using a hydroquinone-induced vitiligo mouse model found that apigenin significantly prevented vitiligo by acting as an anti-inflammatory, increasing tyrosine, and reducing the expression of non-phosphorylated P38 mitogen-activated protein kinases (p38MAPK). The activation of the p38MAPK pathway, which resulted in an increase in melanogenesis in B16 cells, was also reported by Ye et al. [155], who suggested apigenin for hypopigmentation disorder treatment. On the other hand, the glycoside of apigenin, apigenin-6-C-glucoside, tends to suppress melanin synthesis via the down-regulation of intracellular tyrosinase signalling due to the presence of the hydroxyl group at the A and B rings [156].

### Scutellarin

**Figure 4.**
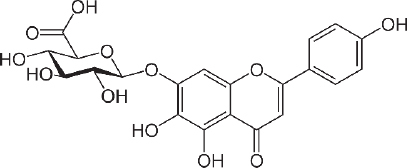
Scutellarin

Scutellarin (7-O-β-D -glucuronide - C_21_H_18_O_12_ - Figure 4) was first investigated for drug development in the late 1970s when it was isolated from the Chinese herbal plant, *Erigeron breviscapus* [157]. This flavonoid is studied for the treatment of heart disease, stroke and diabetes complications because of its ability to relax blood vessels and its anti-inflammatory, antimicrobial, anticoagulation, antioxidant properties and myocardial protection [158,159]. The scutellarin relaxing effect was studied for preventing SARS CoV-2 using molecular docking by Chen and Du [160] and found that scutellarin was able to interact with angiotensin-converting enzyme 2 (ACE2), the host receptor of SARS CoV-2.

The binding energy to ACE2 is estimated at --62.3415 kJ/mol, with binding sites at Glu495, Unk957, and Arg482. For the treatment of stroke, scutellarin was reported to have therapeutic effects on cerebral ischemia by activating the astrocytic Janus kinase 2/signal transducer and activator of transcription 3 (JAK2/STAT3) signalling pathway [161], and by reducing the infarct cerebral tissue area in middle cerebral artery occlusion (MCAO) rats [162].

Luo *et al*. [163] reported that scutellarin, which has anti-inflammatory activity, can provide protection against hyperglycaemia induced by vascular inflammation. Furthermore, Long *et al.* [164], who conducted research on mice, reported that scutellarin was able to inhibit damage to apoptotic cells and disruption of mouse testes morphology due to hyperglycaemia. Su *et al.* [165] also reported the ability of scutellarin to inhibit protein kinase translocation by *in vivo* and *in vitro* studies, making it possible to treat complications due to diabetes. Wang *et al.* [166] reported the protective effect of scutellarin on intervertebral disc degeneration (IVDD) by reducing the amount of ROS, alleviating mitochondrial damage, and decreasing the expression levels of apoptosis-related biomarkers.

The antitumor activity of scutellarin was reported through inhibiting proliferation and inducing apoptosis of HepG2 cells in liver cancer [167], attenuating the development of fibrosarcoma and inhibiting cancer cell metastasis [168], and inhibiting the invasive potential of melanoma cell lines by suppressing the EMT and angiogenesis through the PI3K/Akt/mTOR signalling pathway [169]. Scutellarin was also reported to significantly reduce multiple myeloma xenograft tumour burden in nude mice [170]. Research on white mice reported the protective effect of scutellarin against acute cardiac toxicity due to the use of the drug doxorubicin, one of the most frequently used cancer drugs [171]. Besides that, scutellarin was also able to protect against the disruption of blood flow to the heart [172].

Scutellarin also reports the capability to inhibit cellular tyrosinase enzymes, leading to decreased melanin production with no cytotoxicity effect [173]. Using *in vitro* and computational simulation, Chen *et al.* [174] reported scutellarin inhibited tyrosinase activity in a competitive manner with an *IC*_50_ of 91 μM and predicted that scutellarin was mainly bound with tyrosinase via Arg268 residue.

### Hispidulin

**Figure 5.**
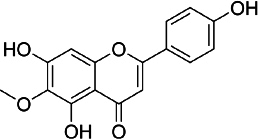
Hispidulin

Hispidulin (4',5,7-trihydroxy-6-methoxyflavone, C_16_H_12_O_6_, Figure 5) is one of the flavonoids that is an active ingredient in Chinese medicine and is reported to have anticancer, anti-inflammatory, and antioxidant activities [175]. The anticancer activity of hispidulin is due to its ability to inhibit the proliferation and metastasis of hepatocellular carcinoma (HCC) cells by activating PPAR-γ [176], suppressing allergic inflammatory reactions by reducing the release of histamine and inflammatory cytokines such as TNF-α and interleukin-4 [177]. Hispidulin is also reported to activate AMP-activated protein kinase (AMPK), thereby suppressing eukaryotic initiation factor 4E-binding protein (4E-BP1) via the rapamycin (mTOR) pathway in glioblastoma multiforme (GBM) cells, one of the most common and deadly types of brain cancer [178]. The same mechanism was also reported in relation to ovarian cancer, where activation of AMPK increased the sensitivity of TNF-related apoptosis-inducing ligand (TRAIL), thereby reducing the protein expression of MCL-1, a group of antiapoptotic proteins [179]. In pancreatic cancer, the target of hispidulin is the vascular endothelial growth factor (VEGF) receptor 2 mediated PI3K/Akt/mTOR signalling pathway in endothelial cells, thereby suppressing pancreatic tumor cell growth and angiogenesis [180]. In studies of hepatoblastoma cancer cells hispidulin was reported to induce apoptosis through mitochondrial dysfunction and inhibition of the PI3K/Akt signalling pathway [181].

The anti-inflammatory effect of hispidulin in neuroinflammation was reported by its capability to increase dopamine levels in the prefrontal cortex of phencyclidine-treated mice and reverse social withdrawal in schizophrenia-1 mutant mice [182].

In an *in vivo* study using epilepsy-gerbil models, hispidulin was shown to reduce seizure suffering with the same effect as diazepam, a drug for anxiety disorders [183]. Hispidulin was also reported to relieve intractable motor tic disorder in a mouse model for hyperdopaminergic states, a condition found in patients with schizophrenia and obsessive-compulsive disorder [184]. The anti-seizure effect of hispidulin was reported to be achieved through suppressing the inflammatory process and activating the mitogen-activated protein kinases A in kainic acid-induced rats [185].

The antidiabetic activity of hispidulin was reported by stimulating glucagon-like peptide-1 secretion and suppressing hepatic glucose production [186]. Hispidulin is also reported to have potential as a therapy in diabetic retinopathy because of its ability to improve high glucose-induced proliferation by reducing the expression of protein kinase, phosphorylated extracellular regulated kinase and VEGF-A, and inhibiting mRNA levels of TNF-α [187]. Molecular docking in this study shows that hispidulin has the highest affinity with VEGF-A and the second highest with TNF-α compared to other modelled compounds. Hispidulin forms hydrogen bonds with Cys5, Asp6 and Glu12 in VEGF-A and Tyr227 and Tyr195 on TNF-α.

### Salvigenin

**Figure 6.**
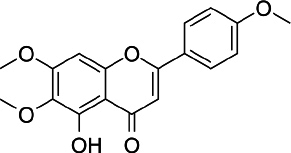
Salvigenin

Salvigenin (5-hydroxy-6,7-bis(trideuteriomethoxy)-2-[4-(trideuteriomethoxy) phenyl] chromen-4-one, C_18_H_16_O_6_, Figure 6) is a derivative of apigenin with different biological activities and reported antioxidant, anticancer and antidiabetic activities [188]. *In vitro* studies using human neuroblastoma SH-SY5Y cells showed that salvigenin protected cells from H_2_O_2_-induced oxidative stress [189], while *in vivo* study using atrazine-induced rats showed that salvigenin protected the liver tissues via regulating antioxidant, anti-inflammatory and anti-apoptotic [190]. Uydes-Dogan *et al.* [191] reported a vasorelaxation effect on rat aortic rings related to antioxidant activity, thus enabling the use of this flavonoid in cardiovascular disease treatment.

Research conducted by Noori *et al.* [192] in female mice showed the antitumor activity of salvigenin through modulation of cytokine production, where there was a decrease in the production of interleukin 4 and an increase in the production of interferon γ. In liver cancer, salvigenin was reported to have the ability to reduce the proliferation, migration and invasion of hepatocellular HCC cells and suppress cell glycosides and chemoresistance by modulating the PI3K/AKT/GSK-3β pathway [193]. Anticancer activity was also reported in relation to oral squamous cell carcinoma, where molecular docking studies showed the binding energy of salvigenin with AKT1 was-33.0536 kJ/mol on Gly294 and Lys179 [194]. The combination of salvigenin with doxorubicin (DOXO), a treatment for chemotherapy patients, was reported to reduce DOXO toxicity through the mechanism of increasing Bax/Bcl-2 ratio, caspase-3 expression and PARP cleavage [195].

Salvigenin was reported to increase insulin secretion and reduce HbA1c, and at the same time was able to influence the lipid profile by reducing triglycerides, total cholesterol, and HDL in diabetic rats [196]. An *in vitro* test using hepatic HuH7 cells shows the capability of salvigenin to inhibit lipogenesis and stimulate mitochondrial functionality [197]. Molecular docking simulation showed that the antidiabetic mechanism of salvigenin is by forming two hydrogen bonds at Lys169 and Glu443 with α-glucokinase [198].

### Acacetin

**Figure 7.**
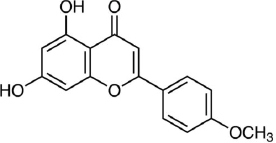
Acacetin

Acacetin (5,7-Dihydroxy-4'-methoxyflavone, C_16_H_12_O_5_, Figure 7) is an apigenin derivative but has different activities from apigenin. Several studies have reported the antioxidant, anti-inflammatory, and anti-cancer activities of this flavonoid [199]. A review by Semwal *et al.* [200]found that there was commercial health supplements based on acacetin with more than 1000 patents related to acacetin as appetite suppression, treatment for prostate cancer, anti-allergy and anti-inflammatory activities. A test on type 2 diabetic mice models shows that acacetin could improve blood glucose and lipid metabolism and liver and kidney dysfunction, where this potential was related to the antioxidant and anti-inflammatory activity of this flavonoid [201]. Using RINm5F cells, Wang *et al.* [202] reported the mechanism of acacetin against lipo-toxicity in pancreatic β-cells. This mechanism involves reducing oxidative stress by scavenging intracellular ROS, upregulating endogenous antioxidant enzymes, diminishing sub-G1 DNA fraction in cells exposed to free fatty acid (FFA), and decreasing endoplasmic reticulum stress by mitigating the overload of intracellular Ca^2+^ and reducing pro-apoptotic protein expression in FFA - stimulated cells. Han *et al.* [203] reported that acacetin has the capability to attenuate diabetes-accelerated atherosclerosis by protecting vascular endothelial cells from injury induced by hyperglycaemia. This protection is achieved by preserving mitochondrial function through Sirt1-mediated activation of Sirt3/AMPK/PGC-1α signalling molecules.

Research related to the anti-inflammatory activity of acacetin has reported the anti-neuroinflammatory effects in Parkinson's disease mouse models. Acacetin was found to protect dopaminergic cells and inhibit the production of inflammatory trigger factors such as nitric oxide, prostaglandin E2, and TNF - α [204]. In the context of dental inflammation, acacetin has shown the ability to suppress inflammation by regulating autophagy and glycogen synthase kinase 3β (GSK-3β) signalling in human periodontal ligament cells [205].

The anticancer activity of acacetin is reported by its ability to inhibit cell growth and induce apoptosis in gastric carcinoma cells [206], inhibit the migration of MDA-MB-231 and T47D cells in breast cancer [207], inhibit the activity of signal transducer and activator of transcription 3 (STAT3) in prostate cancer [208], inhibit the invasion and migration of A549 cells in lung cancer through inhibiting the phosphorylation of Jun N-terminal kinase 1 and 2 (JNK1/2), reduction of activator protein-1 (AP-1) and nuclear factor kappa B - NF-κB [209]. Zhang *et al.* [210] reported that acacetin can induce cell cycle arrest in the G2/M phase, apoptosis, and autophagy in breast cancer cells, resulting in downregulation of PI3Kγ-p110 and the disruption of the PI3K/AKT-mammalian target signalling pathway of rapamycin (mTOR). Molecular docking in this study shows that acacetin forms hydrogen bonds with PI3Kγ via Ser806, Ala885, and Val882, and hydrophobic interactions with Lys833 and Asp964. The PI3K/AKT/mTOR pathway has become a “hot spot” of molecular biomarker-based/targeted therapy because research in several types of cancer, such as breast, liver, colorectal, prostate, and gastric cancers, shows the presence of irregularities in this pathway [211].

### Pectolinaringenin

**Figure 8.**
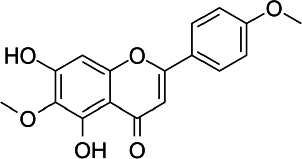
Pectolinaringenin

Pectolinaringenin (5,7-dihydroxy-4′,6-dimethoxy-flavone, C_17_H_14_O_6_, Figure 8) was first isolated from the *Linaria vulgaris* plant more than 100 years ago and has since been isolated as the main component in herbal plants in various countries [212]. Pectolinarigenin has been reported to have various biological activities, such as antioxidant, anticancer, anti-inflammatory, antidiabetic and treatment for various brain-related illnesses [213].

Shiraiwa *et al.* [214] reported the antioxidant activity of pectolinarigenin through *in vitro* inhibition tests using the 2,20-Azobis (2-amidinopropane) dihydrochloride (AAPH) assay. Furthermore, using HepG2 cancer cells, the study reported that pectolinarigenin-induced antioxidant enzymes, heme oxygenase-1, NAD(P)H:quinone oxidoreductase 1, and aldo-keto reductase family 1 member B10. The induction mechanism for these enzymes is through nuclear accumulation of Nrf2 which increases transcriptional activity mediated by the antioxidant response element (ARE) and suppresses Nrf2 degradation through modification of Kelch-like ECH-associated protein 1 (Keap-1). Research by Pang *et al.* [215] using SH-SY5Y neuronal cells showed that pectolinarin, a glycoside of pectolinarigenin can scavenge hydroxyl and nitric oxide radicals, increase cell viability, reduce ROS production and lactate dehydrogenase release (LDH). Therefore, it is promoted to be applied for the treatment of oxidative stress-related neurodegenerative diseases.

The activity of pectolinaringen in inhibiting several types of cancer, including liver cancer, has been reported. Research using HCC cells, SMMC7721 and PLC5, has shown that this flavonoid can suppress the proliferation of HCC cells by inducing cell apoptosis and cell cycle arrest. It also reduces migration and invasion of HCC cells and deactivates the PI3K/AKT/mTOR/ ERK signalling pathway [216]. Research on nasopharyngeal cancer using C666-1 cells have shown that pectolinaringen can induce apoptosis in C666-1 cells through the mitochondrial-related apoptotic pathway and ROS-induced apoptotic pathway [217]. The western blot test in this study showed an increase in cleavage caspase 3 and 9 levels, indicating that the caspase inhibitor (z-VADfmk) significantly prevented the increase of apoptotic cells. Zhou *et al.* [218] reported the potential of pectolinaringen as a treatment for pancreatic cancer using human pancreatic cancer cells (Patu 8988 and BxPC-3). Pectolinaringen induces apoptosis and reduces the phosphorylation of signal transducer and activator of transcription 3 (STAT-3). In breast cancer, *in vitro* assays of MCF-7 cancer cells demonstrated antiproliferative activity of pectolinaringen by inducing apoptosis and downregulation of B-cell lymphoma 2 (Bcl2) expression [219]. Furthermore, research on gastric cancer cells using AGS and MKN28 cells showed the ability of pectolinaringen to inhibit the viability of human gastric cancer cells via the AKT/PI3K/mTOR pathway [220]. This inhibitory mechanism begins with the stimulation of intracellular protein ubiquitination and proteasome degradation of proteins, including caspase-3/-7 and AKT. This is followed by Beclin-1-independent autophagy and subsequent caspase-dependent apoptosis, leading to gastric cancer cells' death.

The anti-melanogenic activity of pectolinarigenin was reported through *in vitro* tests using melan-A cells, where pectolinarigenin was able to inhibit melanogenesis by inhibiting the protein expression of microphthalmia-associated transcription factor (MITF), tyrosinase, tyrosinase-related protein (TRP)-1, and TRP-2, which play a role in synthesis thereby reducing melanin synthesis [221]. In line with its anti-melanogenic ability, Deng *et al.* [222] conducted *in vitro* research using A375 and CHL-1 cells and found that pectolinarigenin was able to inhibit cell viability, proliferation, invasion and migration and induce apoptosis through the apoptotic ROS-mitochondrial pathway.

### Hesperetin and hesperidin

**Figure 9.**
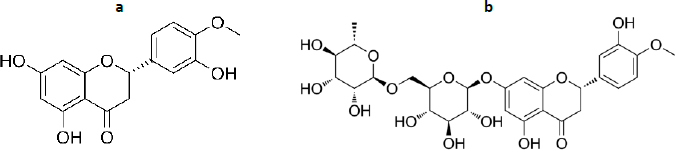
Hesperetin (a), hesperidin (b)

Hesperetin (3',5,7-Trihydroxy-4'-methoxyflavanone, C_16_H_14_O_6_, Figure 9a) together with its glycoside, hesperidin ((2S)-3',5-Dihydroxy-4′-methoxy-7- [α-L- rhamnopyranosyl- (1→6)-β-D-glucopyranosyloxy] flavan-4-one, C_28_H_34_O_15_, Figure 9b) has diverse biological activities, such as antioxidant, anti-inflammatory, neuroprotective, anticancer, cardiovascular protection and antidiabetes [223-225]. The antioxidant activity of hesperetin and hesperidin occurs through direct radical scavenging and augmenting cellular antioxidant defense [226]. The direct radical scavenging pathway plays an important role in protecting the body's DNA, proteins and other tissues. Hesperetin and hesperidin are reported to protect tissue damage from exposure to toxic compounds, such as hydrogen peroxide, 1,2-dimethylhydrazine, benzo(a)pyrene and peroxynitrite [227,228]. In the second pathway, hesperidin is reported to play a role in increasing cellular defence through the ability to upregulate the protein levels of nuclear factor erythroid 2-related factor 2 (Nrf-2) [229]and induction of heme oxidase-1 through extracellular signal-regulated protein kinase [ERK)/Nrf2 signalling [230].

In connection with this antioxidant ability, studies report the inhibitory effect of hesperidin and hespertin on the formation of advanced glycation end products (AGEs), the end products of the Maillard reaction [231]. AGEs cause damage to extracellular proteins, thereby contributing to diabetes complications such as cataracts, nephropathy, vasculopathy, proliferative retinopathy, and atherosclerosis [232]. The study conducted by Shi *et al.* [233] on streptozotocin-induced diabetic rats showed that hesperidin suppressed blood retina breakdown and increased retinal thickness, reduced blood glucose, aldose reductase activity, retinal levels of TNF-α, ICAM-1, VEGF, IL-1β, and AGEs, as well as reduced the level of plasma malondialdehyde (MDA) and increased SOD activity. Using computational molecular docking, Gong *et al.* [234] reported the α-glucosidase inhibition activity of hesperetin, where two hesperetin rings interact with several residues near the active site of the enzyme, such as Lys155, Asn241, Glu304, Pro309, Phe311, and Arg312. An *in vivo* study by Akiyama *et al*. [235]also reported the capability of hesperidin glycosides to reduce blood glucose levels in diabetic rats by altering the activities of glucose-regulating enzymes and lowering the serum and liver lipid levels.

The ability of hesperetin in cancer treatment has been reported in several studies. Yang *et al.* [236] reported the ability of hesperetin as an inhibitor of the transforming growth factor β (TGF-β) signaling pathway, where this inhibition will block the metastatic properties of cancer cells. Through testing on male Wistar rats induced by 1,2-dimethylhydrazine (DMH) as an agent of colon carcinogenesis, Arangathan and Nalini [237] reported that hesperetin was able to reduce tumour multiplicity, tumour incidence and burden of DMH-induced colorectal tumorigenesis. Histological observations showed that the administration of hesperetin in experimental conditions affected colon carcinogenesis at every stage. The group that was given hesperetin continuously showed significant changes compared to the group that was only given hesperetin at the initiation and post-initiation stages. Using the same DMH induction method in male Wistar rats, Nalini *et al.* [238] reported the efficacy of hesperetin as a chemo-preventive agent. It was found to inhibit cell proliferation markers, angiogenic growth factors (VEGF, EGF, bFGF), COX-2 mRNA expression and induce apoptosis. In order to find out the effect of hesperetin on colon cancer apoptosis, the HT-29 human colon adenocarcinoma cell line was used, where hesperetin was able to inhibit the proliferation of HT-29 cells by inducing apoptosis through the Bax-dependent mitochondrial pathway, which involves an oxidant/antioxidant imbalance [239].

In breast cancer research, *in vitro* tests using MCF-7 cells showed that hesperetin inhibited cell proliferation, induced cell cycle arrest at the G1 phase, and induced apoptosis [240]. This study also reports the regulation of cyclin-dependent kinases-4 (CDK-4) and p21^Cip1^, which may participate in the inhibitory mechanism. *In vivo* study reported by Ye *et al.* [241] in a mouse model that was injected with MCF-7aro cells, where hesperetin inhibited the activity of the aromatase enzyme (estrogen synthetase) and suppressed the proliferation of the MCF-7 breast cancer cell line. Aromatase is a key enzyme in the conversion of androgens to estrogen, and exposure to both endogenous and exogenous estrogens has been linked to the initiation and promotion of hormone-dependent diseases such as breast cancer [242]. Measurements of mouse blood plasma showed a decrease in plasma estrogen and messenger RNA expression of the estrogen-responsive gene pS2 in mouse models [241]. This research shows that the mechanism of hesperetin in preventing cell growth is through down-regulating the expression of cyclin D1, CDK4 and Bcl-xL and up-regulating p57^Kip2^ expression.

The skin protective activity of hesperetin tested using murine B16-F10 melanoma cells shows the ability of hesperetin to stimulate melanogenesis through the activation of MAPK, phosphorylation of cAMP-responsive element binding protein (CREB), and glycogen synthase kinase-3b [243]. Using inhibition kinetics and computational simulation, Si *et al*. [244] reported that hesperetin inhibited tyrosinase in a competitive manner and predicted that putative hesperetin-binding residues include Met280, His61, His85, and His259. Another report by Hong *et al*. [245] using multivariate curve resolution-alternate least squares (MCR-ALS) analysis suggested that tyrosinase interacted with hesperetin and formed a tyrosinase-hesperetin complex. Molecular docking showed that hesperetin entered the hydrophobic cavity of tyrosinase PPO and bound near the dinuclear copper active centre. It interacted with Val283, Phe264, His85, Asn260, Val248, and His263 via hydrophobic interactions, formed hydrogen bonds with Met280, His89, and His259 residues, and also interacted with Phe292, His61, Phe90, Glu256, His244, Asn260, Phe264, and Gly281 via van der Waals forces.

In research related to SARS-CoV-2, hesperidin and hesperetin were reported to be able to prevent the binding of the virus to angiotensin-converting enzyme 2 (ACE2) in host cells, inhibit viral replication after penetration into host cells, and prevent and counteract excessive proinflammatory reactions from the immune system [246]. Molecular docking studies on 3 SARS-CoV-2 target proteins, namely SARS-CoV-2 Mpro, SARS-CoV-2 PLpro and SARS-CoV-2 spike glycoprotein, show that the ligand binding affinity score for hesperidin is 24.27, -41.84 and -33.89 kJ/mol, respectively [247]. Another molecular docking carried out on 24 SARS-CoV-2 proteins reported that hesperidin exhibited high binding affinity to 2 target proteins for preventing the virus RNA synthesis and replication (3CLpro and Helicase) and was the only natural compound in the simulation that could target the binding interface between spike and ACE2 through the formation of a hydrogen bond at Tyr440 [248].

### Luteolin

**Figure 10.**
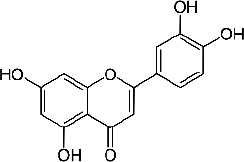
Luteolin

Luteolin (3,4,5,7-tetrahydroxy flavone, C_15_H_10_O_6_, Figure 10) is a natural flavonoid widely isolated from traditional Chinese medicine plants used for treating hypertension, inflammatory disorders, and cancer [249]. Luteolin is reported to have various biological activities such as antioxidant, antidiabetic, neuroprotective, antiallergy, and anticancer [250-252].

Luteolin's antioxidant activity is due to the presence of the 1,4-pyrone moiety group [253] and its ability to donate H atoms with low energy [254]. In *in vivo* study using rat hepatocytes cells induced by tert-butyl hydroperoxide (tBHP), the antioxidant mechanism of luteolin is through up-regulating antioxidant enzyme gene transcription through up-regulating protein heme oxygenase-1, glutamate cysteine ligase, and glutamate-cysteine expression ligase modifier subunit via the extracellular signal-regulated protein kinase 2/nuclear factor erythroid 2-related factor 2 (ERK2/Nrf2) pathway [255].

Antidiabetic activity of luteolin was reported through *in vitro* inhibition tests of the α-glucosidase and α-amylase enzymes [256]. Molecular docking of the α-glucosidase enzyme shows a hydrogen bond between the hydroxyl group of luteolin and Asp285 on H27 and H33 [257], while research by Rekha *et al.* [198] shows a bond energy of -30.9616 kJ/mol with the glucokinase receptor on Ser151, Asp20 and Thr228.

In neuroprotective research, the administration of luteolin improved the learning and memory abilities of Alzheimer's disease model mice. This was followed by the inhibition of neuroinflammation and a decrease in the expression of endoplasmic reticulum stress markers in brain tissue [258]. Research conducted on subarachnoid haemorrhage model mice demonstrated that luteolin was able to repair oxidative damage by increasing the expression of nuclear factor-erythroid 2-related factor 2 (Nrf2) and downregulation of the activation of inflammatory nod-like receptor pyrin domain-containing 3 (NLRP3) [259]. In cases of intracerebral haemorrhage caused by brain injury, luteolin was reported to prevent the activation and infiltration of microglia and reduce the release of proinflammatory factors (IL-6, IL-1β, TNF-α). Additionally, it inhibited the activation of the TLR4/TRAF6/NF-κB signalling pathway [260].

Schomber *et al.* [261] reported an *in vivo* study of luteolin's ability to inhibit the growth of xenografted melanoma tumours in a mouse model induced by tumour cells BRAF-mt A375 and BRAF-wt WM3211 cells. Tumour cells shrank after administration of luteolin, while *in vitro* studies showed luteolin plays a role in the interaction pathway between cells (extracellular matrix), oncogenic pathway, and immune response signalling pathway. In relation to the melanogenesis pathway, an *in silico* study reported the capability of luteolin to inhibit the tyrosinase enzyme by establishing a binding between four hydrogen bonds to Tyr65, Lys79, Cys83, and Glu322 [128]. Molecular docking simulation also shows that the hydroxyl groups of the B ring of luteolin will bind to Asn81 and Cys83, and the HPLC and UPLC-MS analyses explained that luteolin acted as a substrate or a suicide inhibitor [262].

Using human hepatoma cell lines, HepG2, HLF, and HAK-1B, as well as the human neuroblastoma cell line IMR-32, Selvendiran *et al.* [263] reported the protective ability of luteolin against cancer through the mechanism of increasing CD95 (cluster of differentiation 95) expression in neoplastic cells *in vivo* and *in vitro*, promoting phosphorylation of signal transducer and activator of transcription 3 (STAT3) through a ubiquitination-dependent process; and was able to significantly inhibit the growth of human HCC xenografts in nude mice. In esophageal cancer research using human ESCC cell lines (EC1 and KYSE450 ESCC), it was shown that luteolin was able to induce apoptosis and caspase-3 activation as well as induce cell cycle arrest at the G2/M phase, where *in vivo* tests on model mice injected with EC1 showed a decrease in mass tumours in the luteolin treatment group [264].

### Nepetin

**Figure 11.**
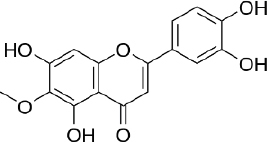
Nepetin

Nepetin (Eupafolin, 6- methoxy-5,7,3′,4′-tetrahydroxy-flavone, C_16_H_12_O_7_, Figure 11) is a flavonoid that was isolated for the first time from *Eupatorium perfoliatum* L., which is widely used in traditional Chinese medicines and Indian tribes [265]. Research shows nepetin has anti-inflammatory, antioxidant and antitumor properties. The anti-inflammatory mechanism of nepetin is to reduce the release of inflammatory mediators (iNOS, COX-2, and NO) and proinflammatory cytokines (IL-6 and TNF-α) in RAW264.7 macrophages induced by lipopolysaccharide [266]. The research also reported inhibition of phosphorylation of p38 MAPK, ERK1/2, JNK, AKT, as well as p65 nuclear translocation of p65 and c-fos.

The antitumor activities of nepetin have been investigated and have revealed significant inhibitory potential on VEGF - induced cell proliferation [267]. Liu *et al.* [268] reported that nepetin inhibited the proliferation of prostate cancer cells by binding to PI3-Akt and attenuating its kinase activity. In a *cervical cancer* study using human cervical adenocarcinoma cells, nepetin was found to induce apoptosis mediated by caspase-dependent pathways involving caspases-3, -9, and -8, which are initiated by the B-cell Lymphoma-2 -dependent loss of mitochondrial membrane potential (Δ*Ψ*_m_) [269]. Chen and Cheng [270] reported the antitumor activity of nepetin in the human non-Hodgkin lymphoma cell line, OCI-LY-3, through inducing cell apoptosis. The study also revealed the ability of nepetin to suppress the Akt/mTOR signalling pathway and promote autophagy.

Xu *et al.* [271]reported the capability of nepetin to inhibit the aggregation of human islet amyloid polypeptide (hIAPP). The research shows the average number of hydrogen bonds between hIAPP22-28 octamer and nepetin increased from 28 to 71, with possible binding sites occurs in Asn22 and Ser28. The hIAPP is the peptide produced by pancreatic β cells in the islet of Langerhans, and the increase of this peptide is associated with type 2 diabetes mellitus [272]. The antidiabetic activity of nepetin was also reported through the inhibition of α-amylase, which was reported to be strong compared to other flavonoids such as scutellarin, apigenin and hispidulin, due to the adjacent position of the dihydroxyl group on the B-ring [273]. The molecular docking simulation of this study shows that nepetin formed four hydrogen bonds with Gln63 and Asp197.

The protective effect of nepetin on the skin was reported by Huey-Ko *et al.* [274], where nepetin was able to decrease cellular melanin content and tyrosinase activity on B16F10 melanoma cells. Inhibition of melanin production occurs through the reduction of phospho-cAMP response element-binding protein and microphthalmia-associated transcription factor (MITF), downregulation of tyrosinase synthesis and TRP expression, and induction of phosphorylation of ERK1/2 and p38 MAPK.

## Conclusions

The isolation and identification of flavonoids from the *Clerodendrum* genus have revealed a variety of compounds using various methods. Several factors determine the flavonoid content in *Clerodendrum* genus, such as the plant material part, the extraction techniques, and the solvent combination. Isolated flavonoids also show significant biological activity, highlighting the antioxidant, anti-inflammatory agents, antimicrobials, antidiabetic, anticancer, anti-tyrosinase, and neuroprotective agents.

## Challenges and perspective

Flavonoids isolated from the *Clerodendrum* genus hold potential benefits for promoting health, but there are several challenges to pursuing their full potential. Future research should focus on optimizing the extraction and isolation techniques, establishing structure-activity relationships, and synthesizing derivatives to enhance the bioavailability and bioactivity of the flavonoids. As research continues to develop the phytochemistry and bioactivities of the *Clerodendrum* genus, there are also opportunities to translate these findings into practical applications, such as the development of functional foods, nutraceuticals, pharmaceuticals, and cosmeceuticals.
